# (*E*)-4-Phenyl­butan-2-one oxime

**DOI:** 10.1107/S1600536811031928

**Published:** 2011-08-11

**Authors:** Hoong-Kun Fun, Wan-Sin Loh, Reshma Kayarmar, Dinesha, G. K. Nagaraja

**Affiliations:** a X-ray Crystallography Unit, School of Physics, Universiti Sains Malaysia, 11800 USM, Penang, Malaysia; b Sequent Scientific Limited, Baikampady, New Mangalore, India; c Department of Chemistry, Mangalore University, Karnataka, India

## Abstract

In the title compound, C_10_H_13_NO, the C—C—C—C torsion angle formed between the benzene ring and the butan-2-one oxime unit is 73.7 (2)°, with the latter lying above the plane through the benzene ring. In the crystal, inter­molecular O—H⋯N hydrogen bonds link pairs of mol­ecules into dimers, forming *R*_2_^2^(6) ring motifs which are stacked along the *a* axis.

## Related literature

For background to oximes and their microbial activity, see: El-Sabbagh *et al.* (1990 ▶); El-Sayed *et al.* (1996 ▶); Althuis *et al.* (1979 ▶); Nargund *et al.* (1992 ▶); Srivastava *et al.* (2004 ▶). For hydrogen-bond motifs, see: Bernstein *et al.* (1995 ▶).
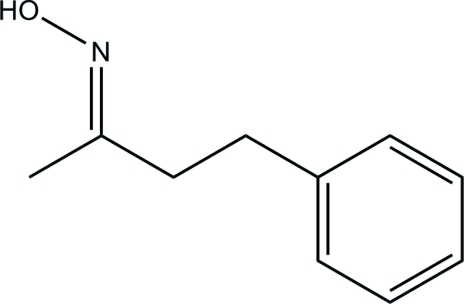


## Experimental

### 

#### Crystal data

C_10_H_13_NO
*M* *_r_* = 163.21
Monoclinic, 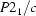

*a* = 5.450 (3) Å
*b* = 9.698 (6) Å
*c* = 18.455 (12) Å
β = 93.888 (13)°
*V* = 973.1 (11) Å^3^
*Z* = 4
Mo *K*α radiation
μ = 0.07 mm^−1^
*T* = 297 K
0.67 × 0.15 × 0.12 mm

#### Data collection

Bruker SMART APEXII DUO CCD area-detector diffractometer
Absorption correction: multi-scan (*SADABS*; Bruker, 2009 ▶) *T*_min_ = 0.953, *T*_max_ = 0.992
10336 measured reflections
2808 independent reflections
1448 reflections with *I* > 2σ(*I*)
*R*_int_ = 0.033

#### Refinement

*R*[*F* ^2^ > 2σ(*F* ^2^)] = 0.053
*wR*(*F* ^2^) = 0.183
*S* = 1.04
2808 reflections
110 parameters
H-atom parameters constrained
Δρ_max_ = 0.18 e Å^−3^
Δρ_min_ = −0.14 e Å^−3^

### 

Data collection: *APEX2* (Bruker, 2009 ▶); cell refinement: *SAINT* (Bruker, 2009 ▶); data reduction: *SAINT*; program(s) used to solve structure: *SHELXTL* (Sheldrick, 2008 ▶); program(s) used to refine structure: *SHELXTL*; molecular graphics: *SHELXTL*; software used to prepare material for publication: *SHELXTL* and *PLATON* (Spek, 2009 ▶).

## Supplementary Material

Crystal structure: contains datablock(s) global, I. DOI: 10.1107/S1600536811031928/tk2777sup1.cif

Structure factors: contains datablock(s) I. DOI: 10.1107/S1600536811031928/tk2777Isup2.hkl

Supplementary material file. DOI: 10.1107/S1600536811031928/tk2777Isup3.cml

Additional supplementary materials:  crystallographic information; 3D view; checkCIF report

## Figures and Tables

**Table 1:** Hydrogen-bond geometry (Å, °)

*D*—H⋯*A*	*D*—H	H⋯*A*	*D*⋯*A*	*D*—H⋯*A*
O1—H1*O*1⋯N1^i^	0.85	1.97	2.785 (3)	160

Symmetry code: (i) 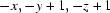.

**Fig. 1.**
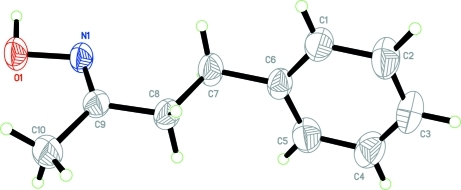
The molecular structure of the title compound, showing 30% probability displacement ellipsoids and the atom-numbering scheme.

**Fig. 2.**
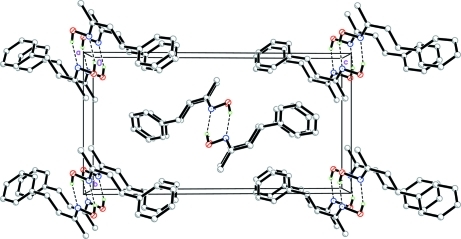
The crystal packing of the title compound, viewed along the a axis.

## supplementary crystallographic information

### Comment

Oximes are important intermediates for the preparation of primary amines by reduction. The primary amine generated can be used for the preparation of many heterocycles like quinoline, azetidinone, 1,2,4-triazole and 1,3,4-thiadiazole, benzothiazipines and thiazolidinone. These heterocycles show various biological activities such as anti-cancer (El-Sabbagh *et al.*, 1990), anti-inflammatory (El-Sayed *et al.*, 1996), anti-allergics (Althuis *et al.*, 1979) anti-microbial (Nargund *et al.*, 1992) and anthelmintic activities (Srivastava *et al.*, 2004). The above motivated us to synthesize the title compound, (*E*)-4-phenylbutan-2-one oxime.

In the title compound (Fig. 1), the torsion angle, C5–C6–C7–C8, formed between the benzene ring (C1–C6) and the butan-2-one oxime (C7–C10/N1/O1) unit is 73.7 (2)°.

In the crystal packing (Fig. 2), pairs of intermolecular O1—H1O1···N1 hydrogen bonds (Table 1) link the molecules into dimers forming *R*_2_^2^(6) ring motifs (Bernstein *et al.*, 1995) which are stacked along the *a* axis.

### Experimental

A mixture of 5-phenylpentan-2-one (2 g, 0.012 mole) and hydroxylamine HCl (1.25 g 0.0184 mole) in ethanol was refluxed for 4 h, during which white crystals separated out. After cooling to room temperature, the resulting (*E*)-4-phenylbutan-2-one oxime was filtered-off, dried and recrystallized from ethanol. Yield, 1.8 g (90%). Crystals suitable for X-ray analysis were obtained from its acetone solution by slow evaporation.

### Refinement

H1O1 was located from the difference Fourier map and was fixed at this position with *U*_iso_(H) = 1.5 *U*_eq_(O) [O–H = 0.8540 Å]. The remaining H atoms were positioned geometrically and refined using the riding model with *U*_iso_(H) = 1.2 or 1.5 *U*_eq_(C) [C–H = 0.93 to 0.97 Å]. A rotating group model was applied to the methyl group.

### Crystal data

**Table:**

C_10_H_13_NO	*F*(000) = 352
*M**_r_* = 163.21	*D*_x_ = 1.114 Mg m^−^^3^
Monoclinic, *P*2_1_/*c*	Mo *K*α radiation, λ = 0.71073 Å
Hall symbol: -P 2ybc	Cell parameters from 1653 reflections
*a* = 5.450 (3) Å	θ = 3.8–22.7°
*b* = 9.698 (6) Å	µ = 0.07 mm^−^^1^
*c* = 18.455 (12) Å	*T* = 297 K
β = 93.888 (13)°	Needle, colourless
*V* = 973.1 (11) Å^3^	0.67 × 0.15 × 0.12 mm
*Z* = 4	

### Data collection

**Table:**

Bruker SMART APEXII DUO CCD area-detector diffractometer	2808 independent reflections
Radiation source: fine-focus sealed tube	1448 reflections with *I* > 2σ(*I*)
graphite	*R*_int_ = 0.033
φ and ω scans	θ_max_ = 30.0°, θ_min_ = 2.4°
Absorption correction: multi-scan (*SADABS*; Bruker, 2009)	*h* = −7→7
*T*_min_ = 0.953, *T*_max_ = 0.992	*k* = −13→13
10336 measured reflections	*l* = −18→25

### Refinement

**Table:**

Refinement on *F*^2^	Primary atom site location: structure-invariant direct methods
Least-squares matrix: full	Secondary atom site location: difference Fourier map
*R*[*F*^2^ > 2σ(*F*^2^)] = 0.053	Hydrogen site location: inferred from neighbouring sites
*wR*(*F*^2^) = 0.183	H-atom parameters constrained
*S* = 1.04	*w* = 1/[σ^2^(*F*_o_^2^) + (0.0833*P*)^2^ + 0.063*P*] where *P* = (*F*_o_^2^ + 2*F*_c_^2^)/3
2808 reflections	(Δ/σ)_max_ < 0.001
110 parameters	Δρ_max_ = 0.18 e Å^−^^3^
0 restraints	Δρ_min_ = −0.14 e Å^−^^3^

### Special details

**Table:**

Geometry. All e.s.d.'s (except the e.s.d. in the dihedral angle between two l.s. planes) are estimated using the full covariance matrix. The cell e.s.d.'s are taken into account individually in the estimation of e.s.d.'s in distances, angles and torsion angles; correlations between e.s.d.'s in cell parameters are only used when they are defined by crystal symmetry. An approximate (isotropic) treatment of cell e.s.d.'s is used for estimating e.s.d.'s involving l.s. planes.
Refinement. Refinement of *F*^2^ against ALL reflections. The weighted *R*-factor *wR* and goodness of fit *S* are based on *F*^2^, conventional *R*-factors *R* are based on *F*, with *F* set to zero for negative *F*^2^. The threshold expression of *F*^2^ > σ(*F*^2^) is used only for calculating *R*-factors(gt) *etc*. and is not relevant to the choice of reflections for refinement. *R*-factors based on *F*^2^ are statistically about twice as large as those based on *F*, and *R*- factors based on ALL data will be even larger.

### Fractional atomic coordinates and isotropic or equivalent isotropic displacement parameters (Å^2^)

**Table:**

	*x*	*y*	*z*	*U*_iso_*/*U*_eq_	
O1	0.0482 (2)	0.64909 (13)	0.46391 (6)	0.0829 (4)	
H1O1	−0.0382	0.5764	0.4564	0.124*	
N1	0.1886 (2)	0.60421 (14)	0.52725 (7)	0.0663 (4)	
C1	0.8076 (3)	0.3977 (2)	0.72018 (9)	0.0761 (5)	
H1A	0.8150	0.3423	0.6793	0.091*	
C2	0.9742 (4)	0.3766 (3)	0.77902 (11)	0.0949 (6)	
H2A	1.0896	0.3062	0.7775	0.114*	
C3	0.9709 (4)	0.4577 (3)	0.83903 (11)	0.0972 (7)	
H3A	1.0844	0.4433	0.8783	0.117*	
C4	0.8004 (4)	0.5602 (3)	0.84143 (10)	0.0977 (7)	
H4A	0.7989	0.6169	0.8821	0.117*	
C5	0.6284 (4)	0.5800 (2)	0.78301 (10)	0.0871 (6)	
H5A	0.5100	0.6486	0.7856	0.104*	
C6	0.6307 (3)	0.49911 (17)	0.72088 (8)	0.0644 (4)	
C7	0.4539 (3)	0.52421 (19)	0.65522 (9)	0.0771 (5)	
H7A	0.2888	0.5351	0.6710	0.093*	
H7B	0.4542	0.4447	0.6233	0.093*	
C8	0.5231 (3)	0.65179 (17)	0.61365 (9)	0.0691 (5)	
H8A	0.5223	0.7300	0.6464	0.083*	
H8B	0.6904	0.6404	0.5997	0.083*	
C9	0.3630 (3)	0.68632 (15)	0.54665 (8)	0.0621 (4)	
C10	0.4212 (4)	0.8158 (2)	0.50706 (11)	0.0922 (6)	
H10D	0.4040	0.7993	0.4557	0.138*	
H10A	0.5870	0.8435	0.5207	0.138*	
H10B	0.3098	0.8874	0.5194	0.138*	

### Atomic displacement parameters (Å^2^)

**Table:**

	*U*^11^	*U*^22^	*U*^33^	*U*^12^	*U*^13^	*U*^23^
O1	0.0914 (9)	0.0805 (8)	0.0714 (7)	−0.0160 (6)	−0.0336 (6)	0.0219 (6)
N1	0.0682 (8)	0.0669 (8)	0.0603 (7)	−0.0095 (6)	−0.0206 (6)	0.0125 (6)
C1	0.0762 (11)	0.0854 (11)	0.0650 (10)	0.0016 (10)	−0.0076 (8)	0.0009 (8)
C2	0.0778 (12)	0.1242 (17)	0.0802 (12)	0.0272 (12)	−0.0129 (10)	0.0050 (11)
C3	0.0823 (13)	0.1379 (18)	0.0681 (11)	0.0064 (13)	−0.0184 (9)	0.0096 (12)
C4	0.1167 (17)	0.1168 (16)	0.0582 (10)	0.0051 (14)	−0.0042 (10)	−0.0083 (10)
C5	0.0883 (13)	0.0952 (13)	0.0767 (11)	0.0192 (11)	−0.0007 (10)	0.0041 (10)
C6	0.0567 (9)	0.0725 (10)	0.0622 (9)	−0.0149 (8)	−0.0088 (7)	0.0149 (7)
C7	0.0691 (10)	0.0795 (11)	0.0787 (11)	−0.0199 (9)	−0.0238 (8)	0.0216 (8)
C8	0.0655 (9)	0.0738 (10)	0.0654 (9)	−0.0189 (8)	−0.0160 (8)	0.0101 (7)
C9	0.0658 (9)	0.0599 (8)	0.0591 (8)	−0.0107 (7)	−0.0068 (7)	0.0047 (6)
C10	0.1152 (16)	0.0778 (12)	0.0808 (12)	−0.0301 (11)	−0.0151 (11)	0.0204 (9)

### Geometric parameters (Å, °)

**Table:**

O1—N1	1.4212 (17)	C5—H5A	0.9300
O1—H1O1	0.8540	C6—C7	1.516 (2)
N1—C9	1.273 (2)	C7—C8	1.517 (2)
C1—C6	1.378 (3)	C7—H7A	0.9700
C1—C2	1.383 (3)	C7—H7B	0.9700
C1—H1A	0.9300	C8—C9	1.502 (2)
C2—C3	1.360 (3)	C8—H8A	0.9700
C2—H2A	0.9300	C8—H8B	0.9700
C3—C4	1.364 (3)	C9—C10	1.497 (2)
C3—H3A	0.9300	C10—H10D	0.9600
C4—C5	1.393 (3)	C10—H10A	0.9600
C4—H4A	0.9300	C10—H10B	0.9600
C5—C6	1.390 (3)		
			
N1—O1—H1O1	98.1	C6—C7—H7A	109.3
C9—N1—O1	112.95 (12)	C8—C7—H7A	109.3
C6—C1—C2	121.40 (18)	C6—C7—H7B	109.3
C6—C1—H1A	119.3	C8—C7—H7B	109.3
C2—C1—H1A	119.3	H7A—C7—H7B	108.0
C3—C2—C1	120.6 (2)	C9—C8—C7	116.60 (13)
C3—C2—H2A	119.7	C9—C8—H8A	108.1
C1—C2—H2A	119.7	C7—C8—H8A	108.1
C2—C3—C4	119.67 (19)	C9—C8—H8B	108.1
C2—C3—H3A	120.2	C7—C8—H8B	108.1
C4—C3—H3A	120.2	H8A—C8—H8B	107.3
C3—C4—C5	119.97 (19)	N1—C9—C10	124.47 (15)
C3—C4—H4A	120.0	N1—C9—C8	118.24 (13)
C5—C4—H4A	120.0	C10—C9—C8	117.29 (14)
C6—C5—C4	121.15 (19)	C9—C10—H10D	109.5
C6—C5—H5A	119.4	C9—C10—H10A	109.5
C4—C5—H5A	119.4	H10D—C10—H10A	109.5
C1—C6—C5	117.14 (16)	C9—C10—H10B	109.5
C1—C6—C7	120.95 (16)	H10D—C10—H10B	109.5
C5—C6—C7	121.86 (17)	H10A—C10—H10B	109.5
C6—C7—C8	111.64 (13)		
			
C6—C1—C2—C3	−1.3 (3)	C1—C6—C7—C8	−103.8 (2)
C1—C2—C3—C4	0.5 (3)	C5—C6—C7—C8	73.7 (2)
C2—C3—C4—C5	1.0 (3)	C6—C7—C8—C9	179.16 (15)
C3—C4—C5—C6	−1.7 (3)	O1—N1—C9—C10	−1.1 (2)
C2—C1—C6—C5	0.5 (3)	O1—N1—C9—C8	179.05 (13)
C2—C1—C6—C7	178.14 (17)	C7—C8—C9—N1	−3.1 (2)
C4—C5—C6—C1	0.9 (3)	C7—C8—C9—C10	177.02 (17)
C4—C5—C6—C7	−176.64 (17)		

### Hydrogen-bond geometry (Å, °)

**Table:**

*D*—H···*A*	*D*—H	H···*A*	*D*···*A*	*D*—H···*A*
O1—H1O1···N1^i^	0.85	1.97	2.785 (3)	160

Symmetry codes: (i) −*x*, −*y*+1, −*z*+1.
